# Flavonol-induced changes in PIN2 polarity and auxin transport in the *Arabidopsis thaliana rol1-2* mutant require phosphatase activity

**DOI:** 10.1038/srep41906

**Published:** 2017-02-06

**Authors:** Benjamin M. Kuhn, Tomasz Nodzyński, Sanae Errafi, Rahel Bucher, Shibu Gupta, Bibek Aryal, Petre Dobrev, Laurent Bigler, Markus Geisler, Eva Zažímalová, Jiří Friml, Christoph Ringli

**Affiliations:** 1 Institute of Plant and Microbial Biology, University of Zurich, Zurich Switzerland; 2 Mendel Centre for Plant Genomics and Proteomics, Central European Institute of Technology (CEITEC), Masaryk University, Kamenice 5, CZ-625 00 Brno, Czech Republic; 3 Institute of Chemistry, University of Zurich, Zurich, Switzerland; 4 Department of Biology – geislerLab, University of Fribourg, Fribourg, Switzerland; 5 Institute of Experimental Botany, Academy of Sciences of the Czech Republic, Prague, Czech Republic; 6 Institute of Science and Technology Austria (IST Austria), Am Campus 1, 3400 Klosterneuburg, Austria

## Abstract

The phytohormone auxin is a major determinant and regulatory component important for plant development. Auxin transport between cells is mediated by a complex system of transporters such as AUX1/LAX, PIN, and ABCB proteins, and their localization and activity is thought to be influenced by phosphatases and kinases. Flavonols have been shown to alter auxin transport activity and changes in flavonol accumulation in the *Arabidopsis thaliana rol1-2* mutant cause defects in auxin transport and seedling development. A new mutation in *ROOTS CURL IN NPA 1 (RCN1*), encoding a regulatory subunit of the phosphatase PP2A, was found to suppress the growth defects of *rol1-2* without changing the flavonol content. *rol1-2 rcn1-3* double mutants show wild type-like auxin transport activity while levels of free auxin are not affected by *rcn1-3*. In the *rol1-2* mutant, PIN2 shows a flavonol-induced basal-to-apical shift in polar localization which is reversed in the *rol1-2 rcn1-3* to basal localization. *In vivo* analysis of PINOID action, a kinase known to influence PIN protein localization in a PP2A-antagonistic manner, revealed a negative impact of flavonols on PINOID activity. Together, these data suggest that flavonols affect auxin transport by modifying the antagonistic kinase/phosphatase equilibrium.

Plant growth and development is influenced and regulated by a network of phytohormones. Among those, auxin is involved in a large number of processes. An important characteristic of auxin is the unequal distribution between cells, which is a prerequisite for cellular differentiation, signaling and cell division. This unequal distribution of auxin is based on polar auxin transport (PAT) between cells and involves the action of auxin transporters^1^,^2^. PAT is mediated by a number of transporters of the AUX1/LAX, PIN, and ABCB class of proteins^3^. ABCBs are mainly apolarly localized and are involved in the long-range auxin transport^4^,^5^,^6^,^7^. PINs often show polar localization, export auxin from cells, and are important for the reflux observed in the root apex resulting in a vectorial auxin stream^8^,^9^,^10^,^11^. Some *pin* loss of function mutants develop strong phenotypes, underlining the importance of PINs for auxin distribution and plant development^12^,^13^. Auxin transport activity is regulated also on the post-translational level by the phosphorylation status of transport proteins. The PINOID (PID) kinase is a central component in this process, which regulates organ development by enhancing PAT and modifying responses to auxin. Consequently, a *pid* mutant shows developmental defects^14^,^15^,^16^. PID kinase activity influences the activity of ABCB1 by phosphorylating the regulatory linker region of this protein^17^. PIN-dependent auxin transport was shown to be regulated by (de-) phosphorylation activity which influences the position as well as the activity of these transporters^18^,^19^,^20^,^21^. The phosphorylation status of PIN proteins in their central hydrophilic loop is determined by the antagonistic activity of the PID kinase and the phosphatase PP2A^22^,^23^. As a consequence, polar auxin transport in roots of *pid* mutants is reduced^24^ while it is increased in the *PP2AA1*-mutant *rcn1 (roots curl in NPA*)^25^,^26^. Mutations in *rcn1*, a regulatory A subunit of PP2A (PP2AA1) or the other two regulatory A subunits PP2AA2 and PP2AA3 influence phosphorylation of PIN proteins, auxin transport, and auxin-related processes^22^.

Flavonoids are a large group of plant secondary metabolites produced via the phenylpropanoid pathway that serve diverse functions in UV protection, pathogen defense, plant-microbe communication, regulation of reactive oxygen species, and auxin transport^27^. Interfering with flavonoid biosynthesis results in defects in root hair and pollen tube growth in petunia and maize^27^,^28^. Flavonols are a subgroup of flavonoids produced by the FLAVONOL SYNTHASE 1, FLS1, and in *Arabidopsis thaliana* consist of kaempferol, quercetin, and isorhamnetin that are glycosylated predominantly by Glc and Rha at the C3 and C7 position, resulting in a large variety of glycosidic forms^29^,^30^. A number of *transparent testa (tt*) mutants were identified based on the pale color of the seed coat due to the absence of proanthocyanidins, a final product of the phenylpropanoid pathway^31^,^32^. Interfering with flavonoid biosynthesis in Arabidopsis by mutating *tt4*, encoding CHALCONE SYNTHASE committed to the initial step in flavonoid biosynthesis, results in altered cycling of PIN1 and increased auxin transport. By contrast, the quercetin-deficient and kaempferol-overaccumulating *tt7* mutant shows inhibited auxin transport^33^,^34^. Flavonols have been shown to compete with the auxin transport inhibitor 1-naphthylphthalamic acid^35^, can inhibit PID kinase activity^17^, and redirect PIN-mediated auxin fluxes^34^,^36^.

The *rol1-2* mutant is affected in rhamnose biosynthesis due to a mutation in the *RHAMNOSE SYNTHASE 1 (RHM1*), resulting in changes in rhamnose-containing pectin and a modified flavonol glycosylation profile with more glucosylated and less rhamnosylated flavonols compared to the wild type. Compared to the wild type, *rol1-2* seedlings develop shorter roots and root hairs, hyponastic instead of epinastic cotyledons, brick-shaped instead of jigsaw-puzzle shaped pavement cells in cotyledons, and deformed trichomes on the first rosette leaves^37^,^38^. Blocking flavonol biosynthesis in the *rol1-2* mutant background by mutations in genes encoding *FLS1* or enzymes in earlier steps of flavonol biosynthesis suppresses the *rol1-2* shoot phenotype, indicating that flavonols present in *rol1-2* interfere particularly with proper shoot development. The root phenotype of the *rol1-2* mutant, in contrast, is only slightly suppressed in the absence of flavonoids, which does not exclude a function of flavonols in root development but suggests that the pectin-induced short-root phenotype of *rol1-2* seedlings is epistatic over the alterations induced by the flavonols^38^,^39^. *rol1-2* mutant shoots show altered auxin signaling and transport activity, effects that are alleviated by preventing flavonol biosynthesis as shown for mutations in *tt4, tt6, fls1*, or *myb111*, the latter being a transcriptional regulator of flavonol accumulation^38^,^39^,^40^. Together, these findings suggest that flavonols present in the *rol1-2* mutant induce alterations in plant development by modifying cellular processes such as auxin transport. Recently, mutations in the flavonol 7-rhamnosyltransferase gene *UGT89C1* were found to suppress *rol1-2*. This suppression, however, is not caused by alterations in the transport but in the levels of auxin metabolites^41^, indicating that flavonols can also influence auxin turnover.

Here, we describe the identification of a suppressor of *rol1-2* that does not influence flavonol accumulation *per se*. The identified *rcn1* mutation alleviates the modified auxin transport activity in *rol1-2*. PIN localization analysis revealed that flavonols in *rol1-2* cause a partial shift in PIN2 polarity and this requires the activity of PP2A. Our data indicate that the flavonols in the *rol1-2* mutant negatively influence the PID kinase activity. Considering the antagonistic activity of PP2A and PID, our work suggests that the *rol1-2* mutant phenotype in part can be explained by the effect of flavonols on the activity of protein kinases such as PINOID.

## Results

### *rcn1* is a suppressor of the flavonol-induced growth defect of *rol1-2* mutant seedlings

**Figure 1:**
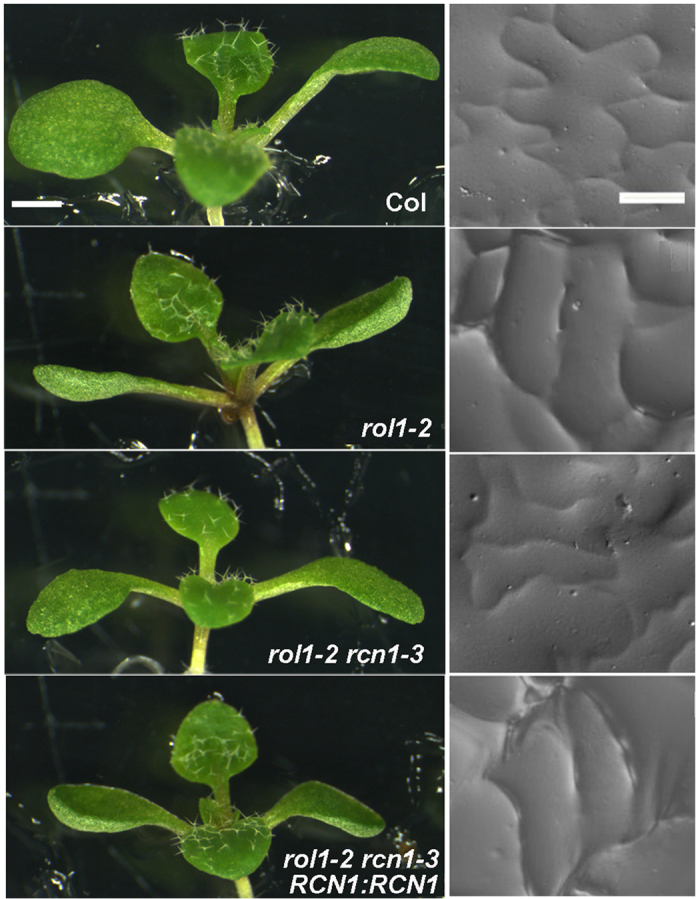
r*cn1-3* is a suppressor of the *rol1-2* phenotype. Arabidopsis wild-type plants show an epinastic bending of cotyledons (left) and jigsaw puzzle-like structure of adaxial epidermal pavement cells (right). The *rol1-2* mutant develops hyponastic cotyledons and distorted, brick-shaped epidermal pavement cells. The *rol1-2 rcn1-3* line shows suppression of the hyponastic growth of cotyledons and partial suppression of the pavement cell shape phenotype. The introduction of an *RCN1:RCN1* genomic clone in a *rol1-2 rcn1-3* line results in the *rol1-2* phenotype. Bar = 1mm (left panels), 40 μm (right panels).

Wild-type Arabidopsis seedlings (ecotype Columbia) develop epinastic cotyledons with puzzle-shaped epidermal pavement cells. By contrast, the *rol1-2* mutant is characterized by hyponastic cotyledons and pavement cells that have lost the typical jigsaw puzzle-like structure (Fig. 1). In addition, roots and root hairs of the *rol1-2* mutant are shorter than those of the wild type. To identify suppressor mutants of *rol1-2*, seeds of this line were mutagenized with ethyl methanesulfonate (EMS) and propagated to the M2 generation^39^. Screening of the M2 seedlings for a suppressed *rol1-2* phenotype resulted in the identification of a number of seedlings with epinastic cotyledons and at least partial re-establishment of the wild-type pavement cell structure. Some of these revealed a strongly reduced flavonol content and were described earlier^39^, whereas others did not show such a reduction.

**Figure 2:**
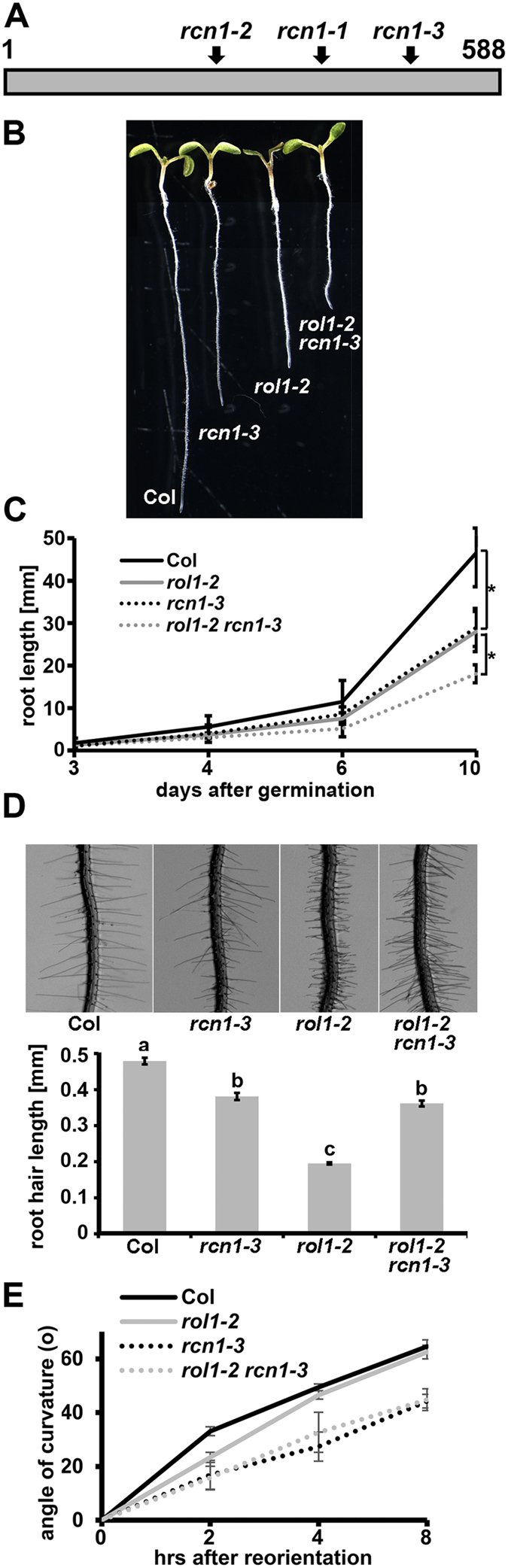
Molecular and phenotypic characteristics of *rcn1-3*. **(A)** Schematic representation of the RCN1 protein with the 15 HEAT repeats. *rcn1-1* and *rcn1-2* are a T-DNA insertion and a 23 bp deletion, respectively, in the Arabidopsis Ws-2 background. *rcn1-3* is an EMS-induced nonsense mutation in the Col background. The mutations are at positions corresponding to K352 (*rcn1-1*), Q251 (*rcn1-2*), and W471 (*rcn1-3*). Numbers relate to amino acid positions in the protein. **(B)** The *rcn1-3* mutation causes a reduction in root length as a single mutant and in the *rol1-2* mutant background. Seven days-old seedlings are shown. **(C)** Graphical representation of root growth of the different lines over a longer period of time. Error bars represent SD, asterisks indicate significant differences (n > 20; t-test; P < 0.05). **(D)** The short root hair-phenotype of the *rol1-2* mutant is alleviated by *rcn1-3*. Root hairs of seven days-old seedlings (top) and the statistical analysis (bottom) are presented. Error bars are shown, different letters on top of the columns indicate significant differences (n = 60; t-test; P < 0.001). **(E)** Gravitropic response after reorientation of agar plates by 90° was measured at three time points. Error bars are shown*, rcn1-3* containing lines are significantly less gravitropic (n > 23; t-test; P < 0.001 after 8hrs).

One recessive, flavonol-accumulating suppressor was mapped to a region on chromosome 1 (for details, see Methods). Since the *rol1-2* mutation is linked to a modification of auxin transport, the genetic interval was searched for potential genes known to influence auxin distribution or function, revealing *ROOTS CURL IN NPA 1 (RCN1*), encoding the regulatory subunit PP2AA1 of the PP2A phosphatase. Sequencing of *RCN1* in the suppressor mutant indeed revealed a point mutation in the coding region introducing a non-sense mutation (Fig. 2A). This mutation is subsequently referred to as *rcn1-3*, the first two *rcn1* alleles having been previously identified in the ecotype Wassilewskija^19^,^42^. The stop codon in *rcn1-3,* introduced in the codon of Trp471, is at the beginning of the 13^th^ of 15 HEAT (Huntingtin-elongation factor 3-phosphatase subunit A-TOR) repeats. *rcn1-1* and *rcn1-2* have T-DNA insertions at positions coding for the 10^th^ and the 7^th^ HEAT repeat, respectively. To confirm that the *rcn1-3* mutation is causing suppression of *rol1-2*, the *rol1-2 rcn1-3* double mutant was complemented with a wild-type copy of *RCN1* and with an *RCN1-GFP* construct under the *RCN1* promoter. Several independent transgenic lines were produced that all showed a *rol1-2* mutant phenotype, confirming that *rcn1-3* causes suppression of *rol1-2* (Fig. 1).

To characterize the *rcn1-3* allele further, an *rcn1-3* single mutant was produced by backcrossing with wild-type Columbia. Similar to the originally identified *rcn1* allele^19^, *rcn1-3* seedlings develop shorter roots than wild-type Columbia, which explains why *rcn1-3* does not suppress the short-root phenotype of *rol1-2* but rather causes a more severe reduction in root length in the *rol1-2 rcn1-3* double mutant, indicative of an additive effect of the two mutations (Fig. 2B,C). By contrast, *rcn1-3* develops a significant portion of wild type-like root hairs which also develop in the *rol1-2 rcn1-3* double mutant (Fig. 2D). Hence, a fraction of the *rol1-2* root phenotype is suppressed by *rcn1-3*. The reduced gravitropic response of *rcn1* reported earlier^25^ is also found for *rcn1-3* compared with Columbia and for the *rol1-2 rcn1-3* double mutant compared to *rol1-2* (Fig. 2E).

### Phosphatase inhibitors allow for chemical complementation of *rol1-2*

**Figure 3:**
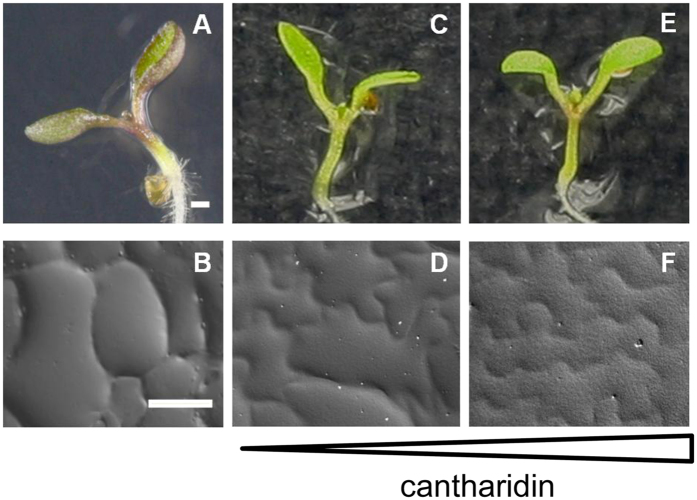
Cantharidin treatment of *rol1-2* plants leads to dosage-dependent suppression of the *rol1-2* phenotype. **(A** and **B)** *rol1-2* mutants show hyponastic cotyledons and defects in pavement cell shape formation. Application of 5 μM Cantharidin on *rol1-2* mutants leads to reduced hyponasty of cotyledons and a partial suppression of the cell shape phenotype **(C** and **D)**. Increasing cantharidin concentration to 10 μM fully suppresses the *rol1-2* phenotype leading to wild type-like bending of cotyledons **(E)** and wild type-like shape of pavement cells **(F)**. Bar = 1 mm (**A,C,E**), 40 μm (**B,D,F**).

The analyses performed so far suggest that the impaired phosphatase activity due to *rcn1-3* suppresses the *rol1-2* phenotype. To corroborate this hypothesis, phosphatase activity in the *rol1-2* mutant was inhibited by pharmaceutical means. Seedlings of the *rol1-2* mutant were germinated and grown in the presence of the phosphatase inhibitor cantharidin, a terpenoid produced by many beetle species that primarily inhibits PP2A activity^43^,^44^ and has been shown to mimic the *rcn1* mutant phenotype^45^. The application of cantharidin led to suppression of the *rol1-2* cell shape phenotype in a dosage dependent manner (Fig. 3). *rol1-2* plants grown on media containing 5 μM cantharidin developed a partial suppression resulting in reduced hyponasty of cotyledons, and a cell shape formation comparable to that of the *rol1-2 rcn1-3* line (Fig. 3C,D). Increasing the cantharidin concentration to 10 μM fully suppressed the cell shape defect and the hyponastic cotyledons, resulting in epinastic cotyledons and epidermal pavement cells that are more similar to the wild type than in the *rol1-2 rcn1-3* mutant (Fig. 3E,F). These findings underpin the genetic data that suggest that reducing phosphatase activity suppresses the *rol1-2* mutant shoot phenotype.

### Flavonol content is not affected by the *rcn1-3* mutation

**Figure 4:**
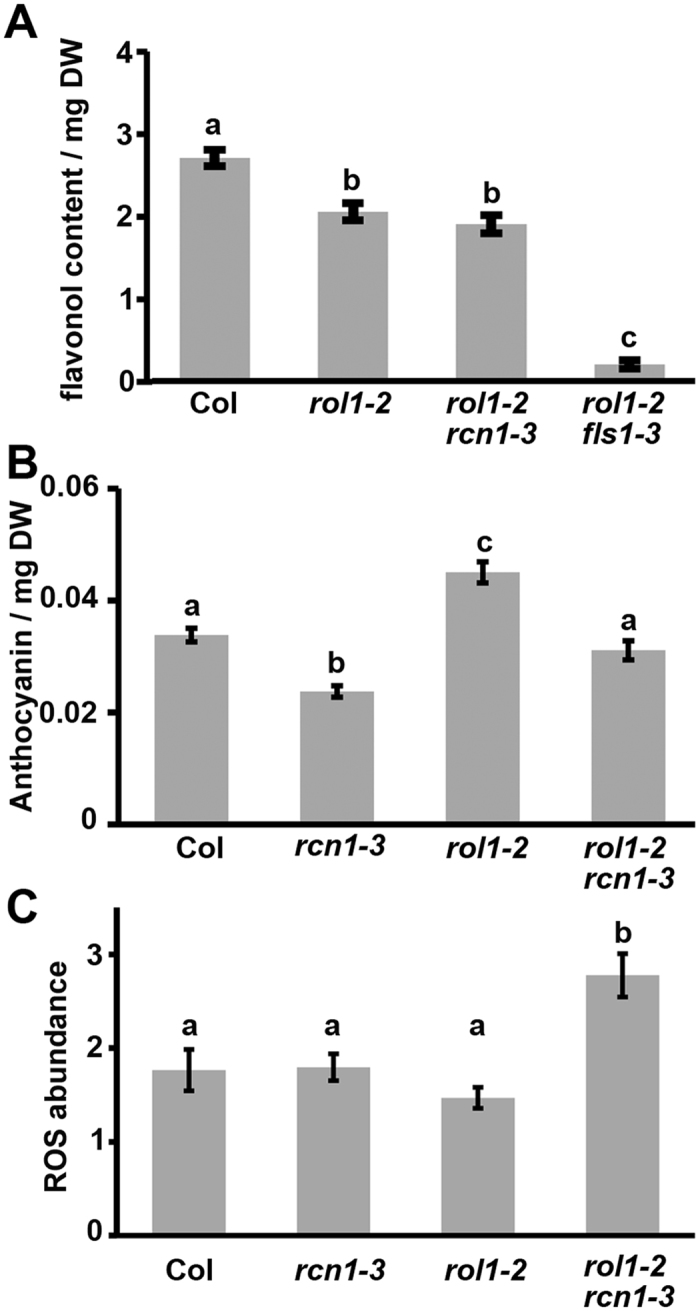
Phenylpropanoid and ROS contents in seedlings. **(A)** *rol1-2 rcn1-3* seedlings show *rol1-2* like flavonol levels in shoots, that correspond to 70% of the wild-type (Col) levels. As a comparison, *rol1-2 fls1-3* mutants are almost devoid of flavonols. Area under the flavonol peaks per mg dry weight was used to quantify flavonol content. **(B)** The anthocyanin content was measured in entire seedlings and revealed to be reduced in the lines containing the *rcn1-3* mutation. **(C)** ROS are quantified by fluorescence emitted by the ROS-specific dye CM-H_2_DCFDA. The tip-area of at least 10 roots was used for each data point. Quantification of ROS revealed comparable levels in the wild type, *rol1-2*, and *rcn1-3* but a significant increase in the *rol1-2 rcn1-3* double mutant. Different letters indicate significant difference between samples (t-test, P < 0.05).

As the *rol1-2* shoot phenotype is strictly dependent on flavonol accumulation, it was important to investigate whether suppression of *rol1-2* by *rcn1-3* was caused by a reduced accumulation of flavonols. To this end, flavonol contents of wild-type, *rol1-2*, and *rol1-2 rcn1-3* seedlings were analyzed by HPLC-MS and signals corresponding to flavonols were identified based on the molecular mass, the comparison with elution profiles done earlier^36^,^38^, and the strong reduction in the flavonol-deficient *rol1-2 fls1-3* double mutant^39^. The area under each flavonol peak was used for relative quantification and the sum of all peak areas represents the relative total amount of flavonols. This revealed that the shoot flavonol content is very similar between the *rol1-2* and the *rol1-2 rcn1-3* mutants and only slightly lower than in the wild type (Fig. 4A). As expected, the *rol1-2 fls1-3* double mutant contained only very low amounts of flavonols. Hence, *rcn1-3* suppresses the *rol1-2* shoot phenotype by a mechanism that does not involve changes in flavonol accumulation.

The paler green appearance of the *rol1-2 rcn1-3* double mutant and the cantharidin-treated *rol1-2* seedlings (Fig. 3) indicated a possible reduction in the anthocyanin content as a cause of impaired phosphatase activity. Anthocyanin quantification in the different plant lines indeed revealed a reduction in the *rcn1-3* containing lines by one third (Fig. 4B). Thus, the later biosynthetic steps of the phenylpropanoid pathway leading to anthocyanin accumulation are reduced by the inhibited phosphatase activity, whereas the enzymatic steps to flavonol biosynthesis are not affected.

Flavonoids are known to scavenge reactive oxygen species (ROS), which function as signaling molecules and influence cell growth. It was tested whether the ROS level is altered in *rol1-2* and whether *rcn1-3* further changes accumulation of ROS. Quantification was done with the fluorescing dye CM-H_2_DCFDA (dichloromethyl derivative of dichlorofluorescein diacetate). To avoid interference with chloroplastic autofluorescence, root tissue was chosen for analysis. As shown in Fig. 4C, the ROS levels of the *rol1-2* and *rcn1-3* single mutants are comparable with the wild type. In the *rol1-2 rcn1-3* mutation, by contrast, ROS levels are increased, indicating a synergistic effect between the two mutations.

### Auxin transport and PIN2 localization, but not free auxin, are altered in *rol1-2* and reverted by *rcn1-3*

**Figure 5:**
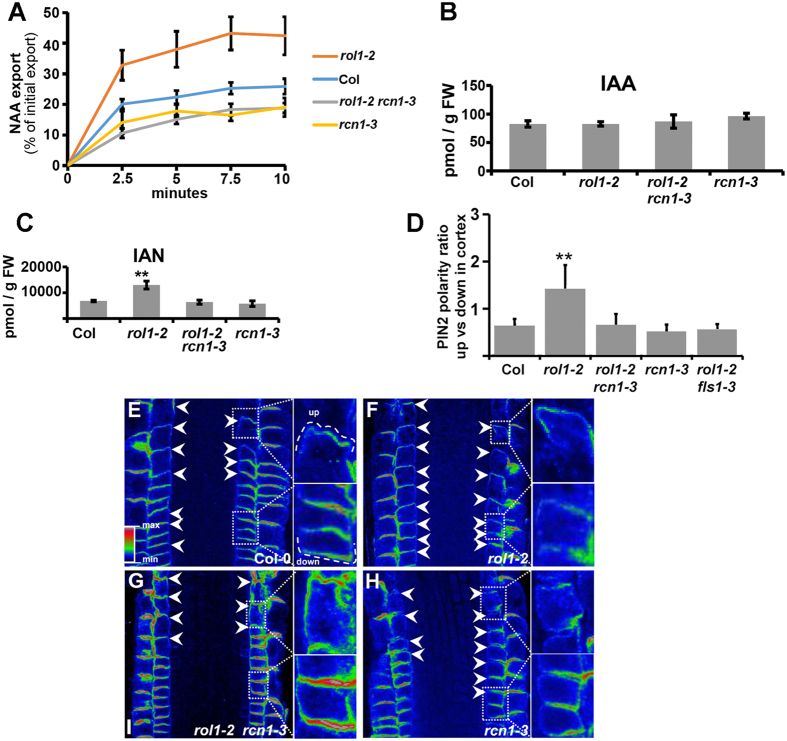
Altered auxin transport and PIN2 localization is altered by *rol1-2* and *rcn1-3*. **(A)** Auxin transport activity was measured by quantifying export of NAA from mesophyll protoplasts. Compared to the wild type (Col), *rol1-2* mutant protoplasts show a significantly increased export activity, which is suppressed by in the *rol1-2 rcn1-3* double mutant. *rcn1-3* and *rol1-2 rcn1-3* are not significantly different from the wild type (N ≥ 4, t-test, p < 0.05). **(B)** The concentration of free auxin is comparable in all four lines, whereas the auxin precursor IAN (indole-3-acetonitrile) is increased in *rol1-2* but at wild-type levels in the other lines **(C)** (t-test, p < 0.05, N = 4; error bars represent SD, double asterisk represents statistically significant difference). **(D)** The graph represents quantification of PIN2 polarity in cortex cells expressed as a ratio of cells showing apical vs basal localization of this auxin efflux carrier in the different genetic backgrounds; error bars represent SE for 5 biological repeats (number of seedlings imaged per each replicate; N > 4; double asterisk represents statistically significant difference (p-value < 0,01). **(E–H)** Confocal images of Arabidopsis root tips after PIN2 immunolocalization reveal greater number of cortex cells showing apicalization of this auxin efflux carrier in the *rol1-2* mutant allele **(F)** in comparison to the control **(E)** and the rescued PIN2 localization in the *rol1-2 rcn1-3* double mutant **(G)** resembling the control line. The *rcn1-3* single mutant showed PIN2 distribution comparable to the wild type **(H)**. Arrowheads indicate apically localized PIN2 in cortex cells. The adjacent insets contain magnifications of cells showing representative apical (up) or basal (down) polarity, for clarity also demarcated with the dashed white line.

Previous analyses revealed a modified auxin transport activity in the *rol1-2* mutant, which was induced by altered flavonol accumulation^39^. This led to the hypothesis that *rcn1-3* suppresses the *rol1-2* shoot phenotype by alleviating the flavonol-induced changes in auxin fluxes without affecting flavonols *per se*. To test this hypothesis, efflux of the synthetic auxin, NAA, was measured in wild-type, *rol1-2*, and *rol1-2 rcn1-3* employing a cellular, protoplast-based auxin transport system^4^. The *rol1-2* mutant showed significantly enhanced auxin export compared to the wild type, confirming previous results^39^. *rol1-2 rcn1-3* protoplasts, however, showed a reduction in auxin transport below but not significantly different from wild-type levels, thus reverting the effect of *rol1-2* on auxin transport. The effect of *rcn1-3* on auxin transport does not depend on *rol1-2* since the *rcn1-3* single and *rol1-2 rcn1-3* double mutants show comparable transport rates (Fig. 5A).

Further, the levels of IAA metabolites, the IAA precursor IAN and different IAA derivatives were determined. The *rcn1-3* mutation does not affect free IAA, which is present to comparable levels in all the lines (Fig. 5B). For most of the auxin metabolites, *rcn1-3* does not have a significant effect, with the exception of the precursor indole-3-acetonitrile (IAN), whose increased level in *rol1-2* is reduced by *rcn1-3*, and the very low-abundant IAA-Glc that is further increased in *rol1-2 rcn1-3* compared to *rol1-2* (Fig. 5C; Supplementary Fig. S1). Thus, the *rcn1-3* mutation not only suppresses the *rol1-2* shoot phenotype but also reverts the effect of the *rol1-2* mutation on auxin transport, while it does not influence levels of free IAA.

Next, it was tested whether the change in auxin transport observed in the protoplast system is also reflected by a change in polar localization of PIN transport proteins in intact tissue. Such an effect is indicated by the mutation in *RCN1* which has been shown to alter PIN localization^22^. PIN proteins were detected by immunolocalization in root tips, the optimal tissue for this type of analysis. In the majority of young wild-type cortex cells, PIN2 shows basal (lower, towards the tip) localization as previously reported^46^, resulting in a ratio of apical:basal localization of PIN2 of <1 (Fig. 5D,E). By contrast, cortex cells of the *rol1-2* mutant showed mainly apically localized PIN2 (and a ratio of apical:basal localization of PIN2 of >1) (Fig. 5D,F). The localization of other PIN proteins was not visibly affected. In *rol1-2 rcn1-3* seedlings, however, apicalization of PIN2 is reverted back and a majority of cortical cells revealed to have PIN2 protein on the basal side comparable to the wild type (Fig. 5D,G). Hence, the *rcn1-3* mutation reverts the altered PIN2 localization in the *rol1-2* mutant. The *rcn1-3* single mutant showed a wild type-like PIN2 localization (Fig. 5D). To test whether the apicalization of PIN2 in *rol1-2* is induced by flavonols, PIN2 was localized in the flavonol-less *rol1-2 fls1-3* double mutant^39^. In this line, similar to *rol1-2 rcn1-3*, PIN2 showed wild type-like, predominant basal localization (Fig. 5D). This supports the hypothesis that apicalization of PIN2 in *rol1-2* is induced by flavonols and that *rcn1-3* counteracts the flavonol-induced alteration in PIN2 localization.

### Flavonols antagonize PID activity *in vivo*

**Figure 6:**
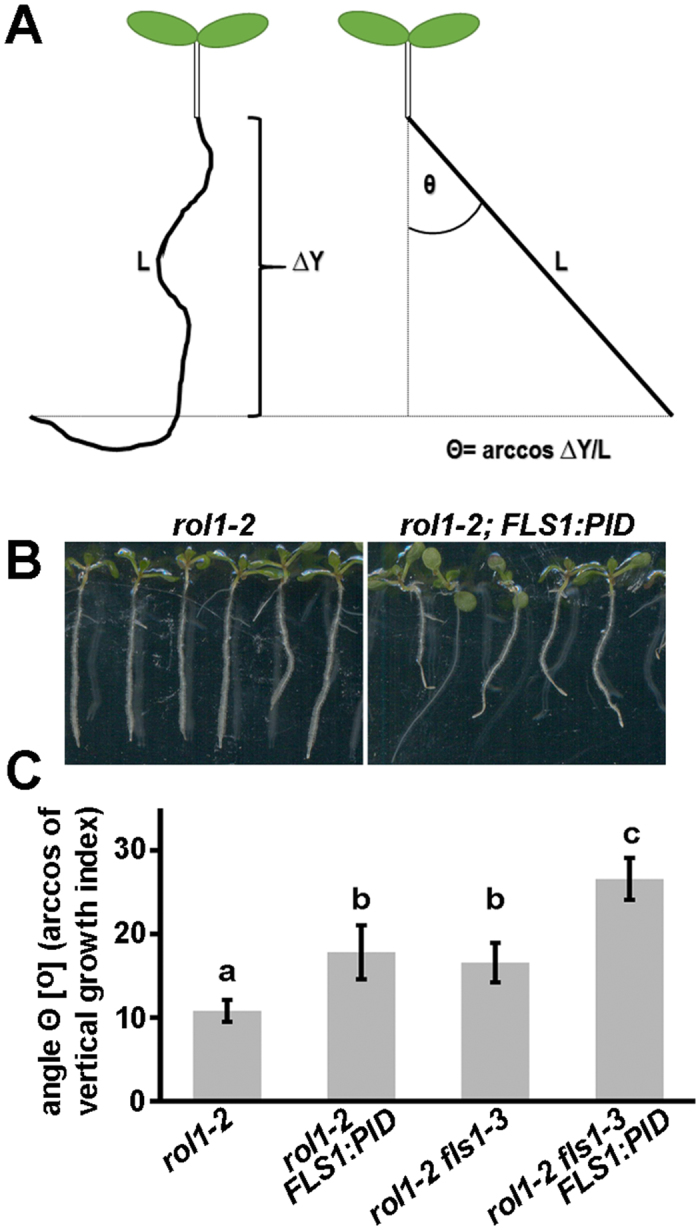
Agravitropism induced by *PID* expressed under the *FLS1* promoter. The angle Θ, corresponding to the arccos of the vertical root growth index ΔY/L **(A)** was used for quantification of the gravitropic response. **(B)** A representative example of seedlings grown for six days in a vertical orientation on MS plates used for quantification of the vertical root growth index. **(C)** The *FLS1:PID* construct induces agravitropism in *rol1-2* and this effect is stronger in the flavonol-less *rol1-2 fls1-3*. Error bars are shown; different letters indicate different significant differences (**C**) (a versus b: P < 0.04; b versus c: P < 0.01; n ≥ 22).

One possible explanation for the opposing effects of the flavonols in *rol1-2* and the *rcn1-3* mutation is that flavonols might reduce kinase activity, while inhibiting PP2A by the *rcn1-3* mutation brings back this antagonistic kinase/phosphatase activity to a balanced state. This hypothesis is supported by the finding of an inhibitory effect of flavonols on PINOID activity *in vitro*^17^. Here, we aimed at testing the effect of flavonols on PID activity *in vivo* by measuring PID-induced agravitropism^16^. To this end, transgenic lines were produced containing the genomic coding region of *PID* under the control of the *FLS1* promoter. In Arabidopsis, *FLS1* codes for the main FLAVONOL SYNTHASE committed to the final step in flavonol biosynthesis and the promoter has been shown to be active in the root in the meristematic region and the elongation and differentiation zones^39^. Hence, these plants express the transgene-encoded *PID* only in tissue competent to accumulate flavonols. Independent transgenic *rol1-2* plants with an *FLS1:PID* insertion at only one genetic locus were crossed with flavonol-less *rol1-2 fls1-3* mutants to have the very same transgenic events in the two backgrounds. The effect of *FLS1:PID* was assessed by analyzing gravitropism using the vertical growth index. This method relates absolute root length to progression of root growth along the Y-axis of plants grown in a vertical orientation and is particularly useful for plants with rather subtle variations of the gravitropic response^47^ (Fig. 6A,B). The value is converted to an angle which is an accurate measurement of gravitropism. As expected, *FLS1:PID* has a clear effect on the gravitropic response in the *rol1-2* mutant which is augmented in the absence of flavonols in *rol1-2 fls1-3*. Already in non-transgenic plants, the absence of flavonols reduces the gravitropic response (Fig. 6C). These data show that *FLS1:PID* has an effect on gravitropism, particularly so in the absence of flavonols, supporting the hypothesis that the flavonols negatively influence PID activity *in vivo*.

## Discussion

The growth defects of the *rol1-2* mutant correlate with altered flavonol accumulation and a change in auxin transport^38^,^41^. The suppression of *rol1-2* by *rcn1-3* indicates a connection between flavonols and PP2A phosphatase activity modulated by the regulatory subunit, RCN1^45^. While *rcn1-3* reduces the accumulation of anthocyanins in the wild type and the *rol1-2* mutant by 30%, the branch of the phenylpropanoid pathway leading to flavonol biosynthesis^29^ is not affected by *rcn1-3*, excluding the possibility that *rcn1-3* suppresses *rol1-2* by blocking flavonol biosynthesis. The reduction in anthocyanin production, on the other hand, is not decisive for the *rol1-2* growth defects as the anthocyanin-less, kaempferol-overaccumulating *rol1-2 tt7* mutant develops a *rol1-2* shoot phenotype^38^,^39^,^48^. The effect of *rcn1-3* on *rol1-2* is largely restricted to the shoot, while in roots, only the short root hair phenotype of *rol1-2* is alleviated whereas the short root phenotype is unchanged. The latter finding is not unexpected as the *rcn1-3* mutant also develops a short root phenotype as found for other *rcn1* alleles^19^.

The opposite effects of *rol1-2* and *rcn1* on root basipetal auxin transport^25^,^41^ provides a possible explanation for *rcn1-3* suppressing the growth defects in *rol1-2* seedlings. This data is confirmed by the transport assay presented here where the effect of *rol1-2* on NAA transport is reversed by *rcn1-3*. Decreased basipetal auxin transport in *rol1-2* roots^41^ seems in conflict with increased auxin export from *rol1-2* protoplasts found here. However, it is important to recall that protoplasts are unpolarized cells that lack the tissue organization of an intact plant. In roots, polar auxin transport can be affected by changes in polarity and activity of auxin transporters, while in protoplasts only the latter can be measured. Despite its inability to always reflect *in vivo* situations, this assay has proven successful in revealing changes in auxin transport capacities^6^,^17^,^39^,^41^. Together, the data indicate that flavonols of *rol1-2* have an effect on auxin transport which is dependent on RCN1 and thus suppressed in the *rol1-2 rcn1-3* double mutant.

**Figure 7:**
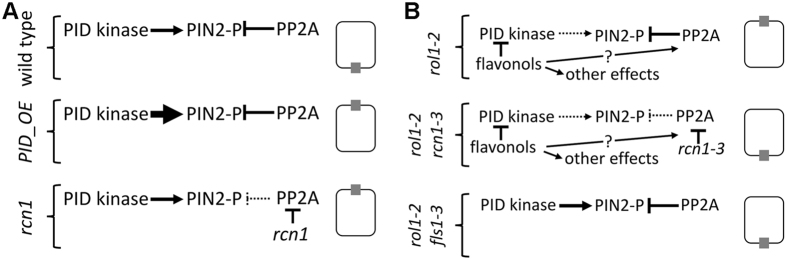
Summary of the effects of flavonols and PP2A on PINOID and PIN2. **(A)** In the wild type, PIN2 preferentially localizes to the basal end of the cell, while PINOID (PID) overexpression or an *rcn1* mutation results in apicalization of PIN2. **(B)** The apicalization of PIN2 in the *rol1-2* mutant background is suppressed by *rcn1-3* (reduces PP2A activity) and by *fls1-3* (blocking flavonol biosynthesis). The flavonols accumulating in the *rol1-2* mutant inhibit PINOID which can be postulated to reduce the PIN2 phosphorylation level and induce a change in PIN2 localization. The *rcn1-3* mutation reduces PP2A phosphatase activity, thus increases PIN2 phosphorylation, and reverts PIN2 localization. For simplicity reasons, PIN2 localization (grey box in a rectangle symbolizing a cell) is overstated and only shown on one side. In reality, a shift in the ratio of basal to apical localization is observed.

The changes in auxin transport in the different mutant lines correlate with a change in polar localization of PIN2. In view of the antagonistic effect of PP2A and PINOID on PIN polar localization^22^, the inhibitory effect of flavonols on PINOID activity^17^ that is confirmed by the *in vivo* experiments presented here, and the comparable effect of a *pinoid* mutant and *rol1-2* on basipetal auxin transport^24^,^41^, a model can be proposed in which the flavonol-induced inhibition of PINOID causes a change in the ratio of basal to apical PIN2 localization and this effect is alleviated by the reduced PP2A activity in *rol1-2 rcn1-3* (Fig. 7). This model fits with the observed changes in PIN2 localization and auxin transport.

Previous work showed that a reduction in PP2A activity induces a basal-to-apical shift of PIN2^20^,^22^,^49^. In the *rol1-2* mutant background, however, *rcn1-3* induces an apical-to-basal shift of PIN2, while it does not have a significant effect on PIN2 localization as a single mutant. Thus, while the proposed model of the *rol1-2* mutant background (Fig. 7B) is sound in itself, it is not entirely agreeing with the current understanding of the regulation of PIN polarity (Fig. 7A)^20^,^22^. This might be because of this particular *rcn1* allele or because mutating only this *PP2AA1* subunit has a weaker effect than mutating two *PP2AA* subunits as previously described^22^. It appears that flavonols in *rol1-2* not only modify PIN2 localization but also change the effect of PP2A on PIN2 localization. Hence, flavonols seem to have different entry points at which they modulate the network controlling auxin transport (Fig. 7B). PIN2 has multiple phosphorylation sites and flavonols might affect kinases other than PINOID which phosphorylate PIN2 and thereby have inverse effects on localization and activity of PIN2. The observed change in PIN2 polar localization is most likely not the only consequence of flavonols and PP2A activity on the auxin transport machinery. Other changes might actually have a stronger impact since it is not plausible why the more apical (shoot-ward) localization of PIN2 in *rol1-2* should result in lower basipetal (shoot-ward) auxin transport^41^. The *pinoid* mutant *pid-9* has a PIN2-dependent effect on auxin transport by changing PIN2 turnover rather than PIN2 polar localization^24^. Also, *rol1-2* and *rcn1-3* have a clear effect in the protoplast assay were cell polarity is lost and where PIN2 is present in very low amounts. Thus, other proteins involved in auxin transport can be influenced by flavonols and PP2A activity and they might be affected in their activity and not their localization, as shown for targets of a family of AGC protein kinases that influence auxin transport^21^,^50^,^51^ or auxin transporters of the ABCB family^6^,^17^.

Flavonols are known scavengers of ROS and thereby can modulate auxin levels^52^,^53^,^54^. The amount of free auxin, however, is comparable among the wild type and the *rol1-2* and *rcn1-3* single mutants. The observation of an increased ROS level only in the *rol1-2 rcn1-3* double mutant indicates a synergistic interaction between these two mutations which is not understood at this point but has no significant effect on the level of free auxin. Previous work has shown that flavonols influence accumulation of different auxin metabolites^41^. The auxin precursor IAN is increased in *rol1-2* and lowered to wild-type levels in the *rol1-2 rcn1-3* double mutant. This increase in IAN, however, is unlikely to be responsible for the growth defects in *rol1-2* as a mutation in the flavonol rhamnosyltransferase *ugt89c1* suppresses *rol1-2* but does not reduce IAN levels^41^. Hence, the results point at auxin transport as the activity that is under the PP2A-dependent influence of flavonols.

## Methods

### Plant material and growth conditions

All plant lines are in the Columbia (Col_0) genetic background. The *rol1-2* and *fls1-3* mutants and the molecular markers for the mutations are described elsewhere^37^,^39^. The polymorphism of the *rcn1-3* allele (G to A substitution at position 2488 relative to the start codon of the genomic sequence) is detected by PCR with the primers *N1759_F* CAGAGGAGTTTGGTCCTCCATG (positions underlined are mutated compared to the wild-type RCN1 sequence) and *N1759_R* CTCAATATTTGCAGCTTTAGTG, followed by digestion with *Nco* I, which only cuts the wild-type sequence.

The identified *rol1-2 rcn1-3* double mutant was back-crossed several times to *rol1-2* and a 1:3 ratio of suppressed versus *rol1-2* phenotype was observed, confirming the recessive nature of *rcn1-3*.

Seeds were surface sterilized (1% sodium hypochlorite, 0.03% TritonX-100) and stratified 2–4 days at 4 °C. Seeds were plated on 1/2 Murashige and Skoog medium (0.6% phytagel (Sigma), 2% sucrose, 100 μg/mL myo-inositol) and grown in growth chambers with 16 h light, 8 h dark cycles at 22 °C.

### DNA constructs and plant transformation

For the *RCN1* complementation construct, the *RCN1* genomic clone was PCR-amplified with the primers *PP2A_CF* CTATAAGACTTGTGATATCAATTG and *PP2A_CR* CTCTTGGAAAATAGGAGATATAAC, encompassing 1.5 kb promoter, 3.2 kb coding sequence (CDS), and 0.5 kb terminator sequence. The resulting fragment (*RCN1:RCN1*) was cloned into *pGEM-T easy* (Promega) for sequencing. For the GFP fusion construct, a *Kpn* I was introduced into *RCN1:RCN1* clone by PCR 3″ of the ATG (N-terminal fusion). PCR was performed with the primers *PP2A_NGFP_F* GGTACCGCTATGGTAGATGAACCG and *PP2A_NGFP_R* GGTACCCATCTTATGTGAAAGTTCG. A previously produced *GFP* construct flanked by *Kpn* I sites was cloned into these *Kpn* I sites, resulting in *RCN1:GFP-RCN1*. These constructs were cloned into the binary vector *pBART27*^55^,^56^ by *Not* I.

For the *FLS1:PID* construct, the *FLS1* promoter was amplified with the primers *FLS1_F3_PC* AATTTCTACTGAATTCGACAGAG and FLS1_Prom_R GGATCCTATTTTTTTTGGTAGTTTGCGTTGC, the FLS1 terminator with the primers *FSL1_Term_F* CTCGAGTGAGAAAAATCAATACGAGAAGAATA and *FLS1_R3C_R* TAATAGCGAATGTGTCGGTTTG, and the PID genomic coding sequence with the primers *PID_FLS_F* GGATCCATGTTACGAGAATCAGAC and *PID_FLS_R* CTCGAGAAAGTAATCGAACGCCGC. The PCR fragments were cloned into *pGEM-T easy* (Promega) for sequencing and correct clones were used to fuse the three fragments in *pGEM-T easy* via the *Bam* HI site at the *FLS1* promoter-*PID* border, and the *Xho* I site at the *PID* -*FLS1* terminator border. A correct construct was cloned into the binary vector *pART27*^56^ by *Not* I.

Plants transformed with the binary vector *pBART27*^55^,^56^ were selected on MS medium containing 100 μg/mL Ticarcillin and 10 μg/mL basta. T2 lines of transgenic lines were grown on basta-containing plates and lines segregating 3:1 for resistance indicating T-DNA integration at one genetic locus were subsequently used for analysis.

### Microscopic analyses

Light microscopic observations were made using a Leica DM6000 stereomicroscope. Imprinting of pavement cells was done as published^57^ and observed with a Zeiss AX10 microscope.

### Root length and gravitropism measurements

For root length quantification, seedlings were grown on MS medium in a vertical orientation. Agar plates were subsequently scanned and root length was determined using ImageJ. For gravitropic response analysis, plants were grown for six days on MS plates, laid out straight on fresh MS plates and incubated in the dark. After 24 hrs, the plates were turned by 90^o^ and then scanned after 2, 4, and 8 hrs. Angles were determined using ImageJ.

### PIN protein localization

Automated whole-mount protein immunolocalization was done utilizing the InsituPro VSi pipetting robot adopting the protocol as described previously^58^. The anti-PIN2 rabbit antibody (kindly provided by Prof. C. Luschnig)^59^ was used at 1:800 dilution. For the secondary antibody, we used Cy3 anti-rabbit (Sigma-Aldrich) in a 1:600 dilution. For immunolocalization seedlings were grown for four days vertically in Petri dishes on 0.8% agar 0.5 Murashige and Skoog (MS; www.sigmaaldrich.com) medium containing 1% sucrose (pH 5.7) at 21 °C, and under a long-day photoperiod unless otherwise indicated. Prior immunolocalization seedlings were fixed with 4% paraformaldehyde as published previously^58^.

Polarity evaluation of was performed by visual inspection of immunolocalized PIN protein localization in cortex cells on acquired confocal pictures. However, during confocal picture acquisition the microscope slides were designated with numbers only to facilitate unbiased picture acquisition. Furthermore, to avoid bias in the polarity determination, all picture sets of all biological repetitions of particular lines (named: Col-0, *rol1-2, rol1-2 x rcn1-3, rcn1-3, rol1-2 x fls1-3*) were saved in folders that were only designated with numbers (not names). Thus the order of samples was not known to the human experimenter evaluating the pictures. Polarity observations were recorded (% of cells with up or down polarity) in Microsoft Office Excel spread sheet and only after the procedure was completed for a particular experiment/biological repeat the sample designations were revealed to the experimenter thus maximizing an unbiased analysis. From this data set, representative pictures were selected for Fig. 5E–G.

### Flavonol and anthocyanin content analysis

The analysis of the flavonol accumulation profile was done as described^39^. Seedlings were grown in a vertical orientation for 6 days as described. One hundred intact seedlings were cut in the hypocotyl region, and roots and shoots were pooled separately, frozen in liquid nitrogen, and lyophilized to determine the dry weight. The dried material was incubated in 500 μL of 80% methanol overnight at 4 °C and subsequently macerated with a pestle, followed by vigorous vortexing. After pelleting the cell debris by centrifugation, the supernatant was transferred to a fresh tube and evaporated in a Speed-Vac centrifuge, with the temperature being limited to a maximum of 42 °C. After evaporation, the pellet was resuspended in 100 μL of fresh 80% methanol and used for analysis. HPLC-ESI-MS and MS/MS experiments were performed on an Acquity UPLC (Waters) connected to a Bruker maX is high-resolution quadrupole time-of-flight mass spectrometer (Bruker Daltonics). An Acquity BEH C18 HPLC column (1.7 μm, 2.1 × 100 mm fitted with a 2 × 2 mm guard column) was used with a gradient of solvent A (H_2_O, 0.1% (v/v) HCOOH) and solvent B (CH_3_CN, 0.1% (v/v) HCOOH), at 0.45 mL flow rate and with a linear gradient from 5 to 95% B within 30 min.

The mass spectrometer was operated in the negative electrospray ionization mode. MS acquisitions were performed in the full scan mode in the mass range from m/z 50 to 2’000 at 25’000 resolution (full width at half maximum) and 2 scans per second. The MS instrument was optimized for maximum signal intensities of quercitrine at *m/z* 447. Masses were calibrated with a 2 mM solution of sodium formate between *m/z* 180 and 1472 prior to analysis. The lock mass signal of hexakis (1H,1H,2H-perfluoroethoxy) phosphazine at *m/z* 556.00195 was further used as lock mass during the HPLC run. Flavonols were identified by the molecular mass determined by MS and by interpretation of fragments obtained by MS/MS, by comparison of both fragmentation patterns and retention times with previous analyses^38^, and the absence in flavonol-less *rol1-2 fls1-3* mutants. The area under each flavonol peak was used for relative quantification since it would be difficult to purify each of the flavonol glycosides in large quantities to produce standard curves. The sum of all peak areas represents the relative total amount of flavonols, which was divided by the amount of plant material used for extraction. These values were compared between different plant lines.

For anthocyanin quantification, 10 days-old seedlings grown on half-strength MS in a vertical orientation were collected, lyophilized, and anthocyanin was extracted by boiling for 10 min in 150 uL of extraction buffer (18% isopropanol, 1% HCl) per mg dry weight. The samples were centrifuged for 5 min and absorption of extracted anthocyanin was measured at 535 nm. Data points represent the mean of biological quadruplicates of each genotype.

### Auxin transport experiments and quantification of derivatives

Arabidopsis mesophyll protoplasts were prepared as described^4^ from rosette leaves of plants grown on soil under 100 μM m^−2^ sec^−1^ white light, 8 h light, 16 h dark cycle at 22 °C. Intact protoplasts were isolated as described^4^ and loaded by incubation with 4-^3^H-1-naphthalene acetic acid (25 Ci mmol^−1^; American Radiolabeled Chemicals) on ice. External radioactivity was removed by separating protoplasts using a 50–30–5% percoll gradient. Samples were incubated at 25 °C and efflux halted by silicon oil centrifugation. Effluxed radioactivity was determined by scintillation counting aqueous phases. Efflux experiments were performed with four to five independent protoplast preparations with four replicas for each time point.

Free auxin, its precursors and metabolites were determined as described^41^ using LC-MS/MS (HPLC - Ultimate 3000 (Dionex, CA, USA) coupled to hybrid triple quadrupole/linear ion trap mass spectrometer (3200 Q TRAP, Applied Biosystems, MA, USA))^60^ after careful extraction^61^ from liquid nitrogen-frozen samples supplied with a mixture of stable-isotope-labelled internal standards: ^13^C_6_-IAA (Cambridge Isotope Laboratories, MA, USA), ^2^H_5_-^15^N_1_-IAA-Asp, ^2^H_5_-^15^N_1_-IAA-Glu, ^2^H_2_-OxIAA (OlChemIm, Olomouc, Czech Republic); 10 pmol each. Quantification was performed using isotope dilution method with multilevel calibration curves (r^2^ > 0.99) and data were processed using the Analyst software (Applied Biosystems).

### ROS quantification

Seven days-old seedlings were incubated in phosphate buffer (pH 7.4) containing 25 μM CM-H_2_DCFDA (chloromethyl derivative of dichlorofluorescein diacetate), a general ROS dye, for 5 minutes followed by three times sequential washing in phosphate buffer to remove excess of the dye. Fluorescent images were taken using Leica SP5 confocal laser-scanning microscope with excitation at 488 nm fluorescence emission at 500–560 nm. Fluorescence was analyzed in epidermal cells of the roots. Individual images (Supplementary Fig. S2) were used for fluorescence intensity analysis using ImageJ software. A square area between elongation zone and root tip was selected for analysis and a bar graph of intensity per unit area for each line was produced.

### Gene identifiers of genes used in this study

FLS1 AT5G08640; RCN1 AT1G25490; ROL1 At1G78570

## Additional Information

**How to cite this article:** Kuhn, B. M. *et al*. Flavonol-induced changes in PIN2 polarity and auxin transport in the *Arabidopsis thaliana rol1-2* mutant require phosphatase activity. *Sci. Rep.* **7**, 41906; doi: 10.1038/srep41906 (2017).

**Publisher's note:** Springer Nature remains neutral with regard to jurisdictional claims in published maps and institutional affiliations.

## Supplementary Material

Supplementary Information
