# Supplementary material for: Flavonol-induced changes in PIN2 polarity and auxin transport in the Arabidopsis thaliana rol1-2 mutant require phosphatase activity
Source: Sci Rep. 2017 Feb 6;7:41906. doi: 10.1038/srep41906 (PMC5292950; doi:10.1038/srep41906)
Supplement: Supplementary Information [file srep41906-s1.pdf]

Flavonol-induced changes in PIN2 polarity and auxin transport in the *Arabidopsis thaliana rol1-2* mutant require phosphatase activity

Benjamin M. Kuhn[1,7], Tomasz Nodzyński[2], Sanae Errafi[1], Rahel Bucher[3], Shibu Gupta[1], Bibek Aryal[4], Petre Dobrev[5], Laurent Bigler[3], Markus Geisler[4], Eva Zažímalová[5], Jiří Friml[6], Christoph Ringli[1#]

Supplementary Figure S1

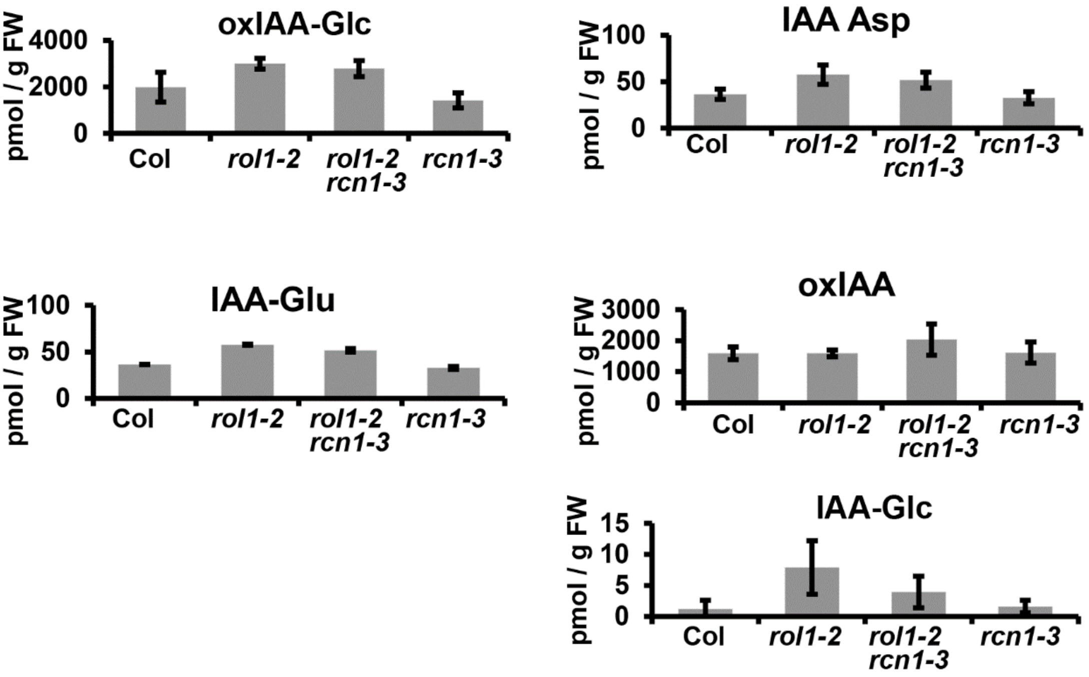

Supplementary Figure S1. IAA metabolites in the different lines. Most of the IAA metabolites are not significantly influences by *rcn1-3*, i.e. *rcn1-3* and wild type or *rol1-2* and *rol1-2 rcn1-3* are comparable. oxIAA: 2-oxindole-3-acetic acid; oxIAA-Glc: 2-oxindole-3-acetyl-glucose (glucosyl-ester of oxIAA); IAA-Glc: indole-3-acetyl-glucose; IAA-Asp: IAA-Aspartate; IAA-Glu: IAA-Glutamate.

Flavonol-induced changes in PIN2 polarity and auxin transport in the *Arabidopsis thaliana rol1-2* mutant require phosphatase activity

Benjamin M. Kuhn[1,7], Tomasz Nodzyński[2], Sanae Errafi[1], Rahel Bucher[3], Shibu Gupta[1], Bibek Aryal[4], Petre Dobrev[5], Laurent Bigler[3], Markus Geisler[4], Eva Zažímalová[5], Jiří Friml[6], Christoph Ringli[1#]

Supplementary Figure S2

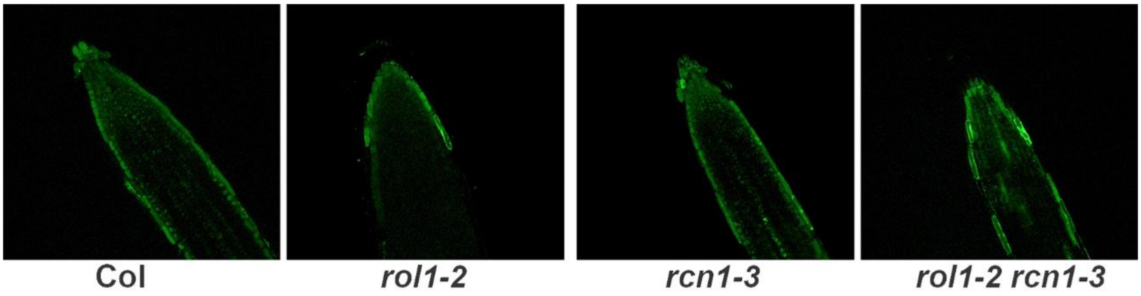

Supplementary Figure S2. Quantification of reactive oxygen species. Pictures of section are shown that were used for quantification of ROS levels in the different lines using CM-H_2DCFDA.
